# MGMT promoter methylation in 1p19q-intact gliomas

**DOI:** 10.21203/rs.3.rs-3393238/v1

**Published:** 2023-10-06

**Authors:** Connor Kinslow, Markus D. Siegelin, Fabio M. Iwamoto, Matthew Gallitto, Alfred I. Neugut, James B. Yu, Simon K. Cheng, Tony J. C. Wang

**Affiliations:** Columbia University

**Keywords:** glioma, MGMT, chemotherapy, 1p19q-intact

## Abstract

**Objective:**

Standard-of-care for 1p19q-intact anaplastic gliomas is defined by the international randomized phase III CATNON trial, which found an overall survival (OS) benefit for adjuvant temozolomide (TMZ) when added to radiotherapy. Paradoxically, TMZ did not appear to benefit patients with IDH-wildtype gliomas, regardless of *MGMT* promoter status. The authors concluded that well-powered prospective study on the clinical efficacy of TMZ for patients with IDH-wildtype anaplastic gliomas (meeting criteria for glioblastoma) is warranted. Given that the prognostic and predictive role of *MGMT* status for grade 2–3 gliomas is unresolved, we determined the effect of *MGMT* status on OS in patients with 1p19q-intact gliomas in the National Cancer Database (NCDB).

**Methods:**

We queried the NCDB from 2018–2019 for patients with IDH-wildtype or -mutant astrocytomas who received chemotherapy with follow-up through 2022. The Kaplan-Meier method and Cox proportional hazards regressions models were used to determine the association of *MGMT* with OS.

**Results:**

We identified 1,514 patients who were newly diagnosed with IDH-wildtype (n = 802, 33% methylated) or - mutant astrocytomas (n = 712, 48% methylated) and received chemotherapy during initial management. An unmethylated promoter was associated with poorer survival in patients with IDH-wildtype (3-year OS 34% [95%CI 29–39%] vs. 46% [95%CI 39–54%], p < .001, adjusted HR 1.53 [95%CI 1.24–1.89]) but not IDH-mutant astrocytomas (3-year OS 79% [95%CI 74–84%] vs. 80% [95%CI 75–86%], p = .81, HR 1.04 [95%CI 0.73–1.50]).

**Conclusions:**

This ancillary analysis supports adjuvant TMZ as standard-of-care for anaplastic astrocytomas (IDH-mutant and 1p19q-intact), irrespective of *MGMT* status. Determining the optimal strategy for diffuse gliomas that are IDH-wildtype will be particularly important. *MGMT* promoter methylation should be considered as a stratification factor in future clinical trials for these patients.

## Introduction

Standard-of-care for 1p19q-intact anaplastic gliomas is defined by the international randomized phase III CATNON trial, which found an overall survival (OS) benefit for adjuvant temozolomide (TMZ) when added to radiotherapy.^1^ The interim analysis did not show a benefit for the concurrent phase of TMZ.^2^ In the initial trial design, isocitrate dehydrogenase (*IDH1/2*) mutational status was not collected and patients were recruited based solely on the absence of 1p19q codeletion, which is observed in approximately 97% of histologically diagnosed (historical) anaplastic astrocytomas and 26% of anaplastic oligodendrogliomas.^3^ Post-hoc analyses of the trial found the benefit of adjuvant TMZ to be limited to the 444 patients with *IDH*-mutant tumors. No benefit was observed in a subset of 159 patients with tumors that met the WHO 2021 molecular criteria for glioblastoma, IDH-wildtype.^4^

O^6^-methylguanine-DNA methyltransferase (*MGMT*) promoter methylation was included as a stratification factor in the CATNON trial, given its predictive role for benefit from TMZ in glioblastoma. Promoter methylation was prognostic in patients with *IDH*-wildtype but not *IDH*-mutant tumors.^4,5^ However, to the surprise of the investigators, *MGMT* status did not predict benefit from TMZ in patients with *IDH*-wildtype tumors. They concluded that a well-powered prospective study on the clinical efficacy of TMZ for patients with anaplastic gliomas who meet the contemporary definition of glioblastoma is warranted. Consistent with CATNON, we also found that *MGMT* status is prognostic in *IDH*-wildtype but not *IDH*-mutant, 1p19q-intact gliomas in a pooled analysis of three prospective cohorts.^6^ *MGMT* status was not an independent predictor of OS on multivariable analysis in either study, though sample sizes in both our study and the CATNON study were limited. Therefore, the prognostic and predictive role of *MGMT* status for grade 2–3 gliomas, particularly IDH-wildtype tumors, is unresolved.

To address this uncertainty, we determined the effect of *MGMT* status on OS in patients with 1p19q-intact gliomas in the National Cancer Database (NCDB) from 2018–2019 with follow-up through 2022. The NCDB accounts for approximately 70% of invasive cancers in the United States, so it is unlikely that inadequate power would lead to ambiguous results.^7^

## Methods

We queried the NCDB (2022 submission) to identify patients with diffuse or anaplastic astrocytoma (International Classification of Diseases (ICD)-O-3 codes 9400 or 9401), IDH-mutant (Brain Molecular Markers 1 or 2) or IDH-wildtype (2 or 4) newly diagnosed between January 1, 2010 and December 31 2019 with follow-up through December 31, 2022. Patients were included if they received chemotherapy. Patients were excluded if they had less than one month of follow-up or missing data for age, sex, race, Charlson-Deyo Comorbidity Index, extent of resection, receipt of radiotherapy, or *MGMT* promoter methylation status.^7-15^

All statistical analyses were conducted using the RStudio software Version 1.4.1106 (RStudio, Inc., Boston, Massachusetts). The Kaplan-Meier method with the log-rank test and adjusted Cox proportional hazards regressions models were used to determine the association of *MGMT* status with OS. Variables that were statistically significant on univariable analysis were included in the multivariable model. Schoenfeld’s test of weighted residuals was utilized to assess proportional hazard assumption in the Cox model. All analyses were performed at the .05 significance level based on two-sided statistical testing.

## Results

**Figure 1:**
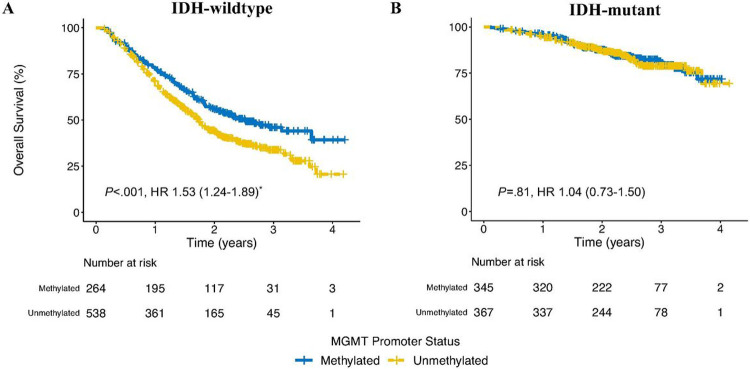
Kaplan-Meier curves for overall survival based on *MGMT*promoter status in patients with IDH-wildtype (A) and IDH-mutant (B) astrocytomas. 95% confidence interval are included in parentheses. *Age, sex, Charlson-Deyo Comorbidity Index, and extent of resection were included in the final multivariable model. HR- Hazard ratio

We identified 2,793 patients who were newly diagnosed with IDH-wildtype or -mutant astrocytomas and received chemotherapy during initial management. We excluded patients with unknown *MGMT* status (44.9%) or other missing data (1.6%). There were 1,514 patients (802 IDH-wildtype and 712 IDH-mutant) included in our final analysis. *MGMT* promoter methylation was observed in 33% and 48% of patients with IDH-wildtype and -mutant astrocytomas, respectively (Table 1). An unmethylated promoter was associated with poorer survival in patients with IDH-wildtype (3-year OS 34% [95%CI 29–39%] vs. 46% [95%CI 39–54%], p < .001, adjusted HR 1.53 [95%CI 1.24–1.89]) but not IDH-mutant astrocytomas (3-year OS 79% [95%CI 74–84%] vs. 80% [95%CI 75–86%], p = .81, HR 1.04 [95%CI 0.73–1.50], Fig. 1). Similar results were observed in a sensitivity analysis that included low methylation (hypomethylated, partial methylation) in the methylated group (3-year OS 32% [95%CI 27–37%] vs. 47% [95%CI 41–54%], p < .001 for IDH-wildtype and 77% [95%CI 71–83%] vs. 82% [95%CI 77–86%], p = .13 for IDH-mutant).

## Discussion

This ancillary analysis supports adjuvant TMZ as standard-of-care for anaplastic astrocytomas (IDH-mutant and 1p19q-intact), irrespective of *MGMT* status. Determining the optimal strategy for anaplastic astrocytomas that are IDH-wildtype (including those that meet the molecular criteria for glioblastoma) will be particularly important. The largest randomized trials (CATNON, RTOG 9402, EORTC 26951, RTOG 9802), paradoxically, have not demonstrated an improvement in OS with alkylating chemotherapy for patients with IDH-wildtype grade 2–3 gliomas, though these were post-hoc analyses with modest sample sizes.^1,2,16-18^ Based on our results, *MGMT* promoter methylation should be considered as a stratification factor in future clinical trials of patients with IDH-wildtype gliomas.

Limitations of our study include retrospective analysis with relatively short follow-up. To clearly determine if *MGMT* promoter methylation may be a prognostic or predictive biomarker in IDH-mutant tumors, longer follow-up is required in both our dataset and the CATNON trial, as approximately 80% and 66% of patients were still alive at the database lock. However, we are not aware of any prospective studies that support a role for *MGMT* status in IDH-mutant astrocytomas. In contrast, emerging data suggest a role for *MGMT* as a biomarker in 1p19q-codeleted oligodendrogliomas.^6,7^ The method of testing for *MGMT* promoter methylation is not reported in the NCDB, and results and cutoff levels may vary depending on the specific assay. Considering these limitations, our study is the largest on the subject and our results are consistent with other literature. Collectively, these data support a role for *MGMT* testing in IDH-wildtype gliomas and IDH-mutant/1p19q-codeleted oligodendrogliomas but not IDH-mutant/1p19-intact astrocytomas.

## Figures and Tables

**Table 1:** Patient Demographic and Clinical Characteristics

Characteristics	IDH-wildtype astrocytoma (*N* = 802)	IDH-mutant astrocytoma (*N* = 712)
Age^1^	60 (47, 68)	37 (30, 49)
Sex	995 (60%)	554 (58%)
Male	442 (55%)	413 (58%)
Female	360 (45%)	299 (42%)
Race		
White	706 (88%)	624 (88%)
Black	46 (5.7%)	35 (4.9%)
Asian/Pacific Islander	20 (2.5%)	18 (2.5%)
Other/Unknown	30 (3.7%)	35 (4.9%)
Charlson-Deyo Comorbidity Index		
0	625 (78%)	609 (86%)
1	99 (12%)	63 (8.8%)
2 or more	78 (9.7%)	40 (5.6%)
Grade		
2	277 (35%)	207 (29%)
3	525 (65%)	505 (71%)
Extent of Resection		
No surgery/Subtotal	607 (76%)	419 (59%)
Gross-total	195 (24%)	293 (41%)
Radiotherapy		
No	22 (2.7%)	31 (4.4%)
Yes	780 (97%)	681 (96%)
*MGMT* Promoter Status		
Methylated	264 (33%)	345 (48%)
Unmethylated	538 (67%)	367 (52%)

1 Median (Interquartile Range)
